# Supplementary material for: Multi‐dataset identification of innovative feature genes and molecular mechanisms in keratoconus
Source: J Cell Mol Med. 2024 Sep 19;28(18):e70079. doi: 10.1111/jcmm.70079 (PMC11412914; doi:10.1111/jcmm.70079)
Supplement: Supplementary file 2 — Material S2. [file JCMM-28-e70079-s002.zip › 7.GSEA analysis/1.ARL11/KEGG_INTESTINAL_IMMUNE_NETWORK_FOR_IGA_PRODUCTION.html]

Details for gene set KEGG\_INTESTINAL\_IMMUNE\_NETWORK\_FOR\_IGA\_PRODUCTION[GSEA]

|  || Dataset | ARL11.group.cls#h\_versus\_l |
| Phenotype | group.cls#h\_versus\_l |
| Upregulated in class | l |
| GeneSet | KEGG\_INTESTINAL\_IMMUNE\_NETWORK\_FOR\_IGA\_PRODUCTION |
| Enrichment Score (ES) | -0.34049034 |
| Normalized Enrichment Score (NES) | -0.91751534 |
| Nominal p-value | 0.5694165 |
| FDR q-value | 1.0 |
| FWER p-Value | 1.0 |
Table: GSEA Results Summary

  

Fig 1: Enrichment plot: KEGG\_INTESTINAL\_IMMUNE\_NETWORK\_FOR\_IGA\_PRODUCTION      
 Profile of the Running ES Score & Positions of GeneSet Members on the Rank Ordered List

  

| SYMBOL | TITLE | RANK IN GENE LIST | RANK METRIC SCORE | RUNNING ES | CORE ENRICHMENT || 1 | ITGA4 | na | 272 | 0.550 | 0.0750 | No |
| 2 | HLA-DRB5 | na | 2778 | 0.251 | 0.0407 | No |
| 3 | HLA-DOA | na | 3812 | 0.207 | 0.0421 | No |
| 4 | CCR10 | na | 4569 | 0.180 | 0.0474 | No |
| 5 | PIGR | na | 6058 | 0.136 | 0.0250 | No |
| 6 | IL6 | na | 6613 | 0.121 | 0.0273 | No |
| 7 | CXCR4 | na | 7115 | 0.109 | 0.0293 | No |
| 8 | HLA-DRB4 | na | 7218 | 0.107 | 0.0425 | No |
| 9 | HLA-DRA | na | 7656 | 0.099 | 0.0448 | No |
| 10 | HLA-DPA1 | na | 7767 | 0.097 | 0.0562 | No |
| 11 | HLA-DRB1 | na | 7965 | 0.093 | 0.0645 | No |
| 12 | CD86 | na | 8057 | 0.091 | 0.0757 | No |
| 13 | HLA-DQB1 | na | 8352 | 0.086 | 0.0801 | No |
| 14 | ICOSLG | na | 8484 | 0.083 | 0.0888 | No |
| 15 | ICOS | na | 10447 | 0.054 | 0.0405 | No |
| 16 | ITGB7 | na | 10543 | 0.053 | 0.0457 | No |
| 17 | IL15RA | na | 10561 | 0.053 | 0.0532 | No |
| 18 | TNFSF13B | na | 11811 | 0.038 | 0.0230 | No |
| 19 | HLA-DPB1 | na | 14439 | 0.016 | -0.0504 | No |
| 20 | IL15 | na | 14630 | 0.014 | -0.0538 | No |
| 21 | HLA-DMA | na | 17342 | -0.006 | -0.1311 | No |
| 22 | HLA-DOB | na | 17625 | -0.008 | -0.1381 | No |
| 23 | CD28 | na | 19606 | -0.021 | -0.1920 | No |
| 24 | HLA-DRB3 | na | 19647 | -0.021 | -0.1899 | No |
| 25 | TNFSF13 | na | 20555 | -0.028 | -0.2119 | No |
| 26 | TNFRSF13C | na | 20657 | -0.029 | -0.2105 | No |
| 27 | CCL28 | na | 22053 | -0.040 | -0.2446 | No |
| 28 | LTBR | na | 22485 | -0.045 | -0.2503 | No |
| 29 | HLA-DQA2 | na | 24037 | -0.063 | -0.2855 | No |
| 30 | AICDA | na | 24863 | -0.074 | -0.2981 | No |
| 31 | CCL25 | na | 25455 | -0.082 | -0.3028 | No |
| 32 | TGFB1 | na | 25459 | -0.082 | -0.2905 | No |
| 33 | CCL27 | na | 26351 | -0.096 | -0.3017 | No |
| 34 | CD40 | na | 26388 | -0.096 | -0.2883 | No |
| 35 | HLA-DMB | na | 27501 | -0.119 | -0.3024 | No |
| 36 | MAP3K14 | na | 28361 | -0.140 | -0.3061 | No |
| 37 | TNFRSF17 | na | 29554 | -0.174 | -0.3143 | Yes |
| 38 | CXCL12 | na | 30202 | -0.194 | -0.3036 | Yes |
| 39 | IL2 | na | 30766 | -0.213 | -0.2878 | Yes |
| 40 | HLA-DQA1 | na | 30949 | -0.219 | -0.2601 | Yes |
| 41 | MADCAM1 | na | 31242 | -0.229 | -0.2339 | Yes |
| 42 | CD80 | na | 31382 | -0.235 | -0.2026 | Yes |
| 43 | CD40LG | na | 31605 | -0.244 | -0.1722 | Yes |
| 44 | IL5 | na | 32563 | -0.292 | -0.1558 | Yes |
| 45 | IL10 | na | 32834 | -0.310 | -0.1169 | Yes |
| 46 | CCR9 | na | 33135 | -0.332 | -0.0755 | Yes |
| 47 | IL4 | na | 33709 | -0.380 | -0.0347 | Yes |
| 48 | TNFRSF13B | na | 34040 | -0.425 | 0.0198 | Yes |
Table: GSEA details [plain text format]

  

Fig 2: KEGG\_INTESTINAL\_IMMUNE\_NETWORK\_FOR\_IGA\_PRODUCTION      
 Blue-Pink O' Gram in the Space of the Analyzed GeneSet

  

Fig 3: KEGG\_INTESTINAL\_IMMUNE\_NETWORK\_FOR\_IGA\_PRODUCTION: Random ES distribution      
 Gene set null distribution of ES for **KEGG\_INTESTINAL\_IMMUNE\_NETWORK\_FOR\_IGA\_PRODUCTION**
